# Decoding positive selection in *Mycobacterium tuberculosis* with phylogeny-guided graph attention models

**DOI:** 10.1186/s12859-026-06583-0

**Published:** 2026-08-06

**Authors:** Linfeng Wang, Susana Campino, Taane G. Clark, Jody E. Phelan

**Affiliations:** 1 Department of Infection Biology, Faculty of Infectious and Tropical Diseases, London School of Hygiene & Tropical Medicine, London, WC1E 7HT UK; 2 Faculty of Epidemiology and Population Health, London School of Hygiene & Tropical Medicine, London, WC1E 7HT UK

**Keywords:** Tuberculosis, Mycobacterium tuberculosis, positive selection, drug resistance, phylogeny, Graph Attention Networks

## Abstract

**Motivation:**

Positive selection is a key evolutionary force in *Mycobacterium tuberculosis*, driving the emergence of adaptive mutations that influence drug resistance, transmissibility, and virulence of tuberculosis. Phylogenetic trees capture the hierarchical evolutionary relationships among isolates, making them an ideal framework for detecting such adaptive signals. Here, we present a phylogeny-guided graph attention network approach, coupled with a novel method for converting SNP-annotated phylogenetic trees into graph structures suitable for graph neural network processing.

**Results:**

Using a dataset of 1000 *M. tuberculosis* isolates, representing the four main lineages, and 206 single-nucleotide variants (75 resistance-associated and 131 neutral) spanning 79 drug-resistance genes, we constructed graphs where nodes represented individual isolates and edges reflected phylogenetic distances. To reduce noise and highlight local evolutionary structure, we pruned edges between isolates separated by more than seven internal nodes. Node features were encoded as binary indicators of SNP presence or absence, and the graph attention network (GAT) architecture comprised two attention layers with a residual connection, followed by global attention pooling and a multilayer perceptron classifier. The model achieved higher accuracy (0.81, AUC 0.82) than a homoplasy-count baseline (0.71) on the held-out test set. Under repeated cross-validation, however, F1 scores did not differ significantly (0.56 vs. 0.58; paired Wilcoxon *p* = 0.98), reflecting a specificity-sensitivity trade-off: the GAT favoured specificity (0.89), the baseline favoured sensitivity. Applying the model to 138 WHO-classified “uncertain” variants identified 28 high-confidence candidates, including *rpoC* Ile491Val (rifampicin, compensatory), *ubiA* Arg240Cys (ethambutol), and *rpsA* Ala412Val (pyrazinamide). These findings demonstrate the feasibility of encoding phylogenetic trees as graph neural network-compatible structures and the utility of attention-based models for detecting positive-selection signals, supporting genomic surveillance and prioritisation of candidates for experimental validation.

**Availability and Implementation:**

The source code for the model can be found at https://github.com/linfeng-wang/Phylogeny-Guided_GAT.

**Supplementary Information:**

The online version contains supplementary material available at https://doi.org/10.1186/s12859-026-06583-0.

## Introduction

Tuberculosis (TB), caused by *Mycobacterium tuberculosis*, remains one of the leading causes of infectious disease mortality worldwide, responsible for 1.23 million deaths in 2024 [1]. The development of drug resistance has exacerbated this epidemic, with 400,000 of all new TB cases reported to have resistance to at least the first-line drug rifampicin. A key driving force underpinning such an increase in the incidence of drug-resistant TB spread is the evolution of mutations in drug targets and pro-drug activators. In the presence of a drug, a selective pressure is exerted on the bacteria, which causes resistance-conferring mutations to rapidly reach fixation in the population. *M. tuberculosis* has a genome of approximately 4.4 Mb [2], with an estimated substitution rate of between 2 × 10^–10^ and 3 × 10^–10^ substitutions per site per generation, equivalent to approximately 0.2–0.5 single-nucleotide polymorphisms (SNPs) per genome per year [3]. *M. tuberculosis* evolves through the accumulation of random mutations, some of which confer a fitness advantage and are subsequently enriched in the population by natural selection. These positively selected mutations may reflect evolutionary responses to drug pressure, host immunity, or transmission dynamics [4], [5]. Importantly, the evolutionary trajectory of *M. tuberculosis* is shaped by its strictly clonal population structure, with no recombination [6]. This allows for the reconstruction of its global phylogeny into distinct lineages [7], each representing deep evolutionary splits. These lineages not only mirror the geographic and demographic history of TB spread, but also capture lineage-specific patterns of drug resistance, virulence, and transmissibility.

Phylogenetic trees are graphical representations of the evolutionary relationships among a set of organisms or genetic sequences, inferred from genetic, genomic, or phenotypic data [8]. In the context of TB, phylogenies help to reconstruct the ancestral relationships between *M. tuberculosis* isolates, often reflecting historical transmission events and the lineage-based population structure [9]. These trees are typically rooted, bifurcating structures in which branch lengths may reflect genetic divergence or time elapsed since a common ancestor. This structure provides a natural framework for studying evolutionary signals, as it explicitly captures the hierarchical ancestry between *M. tuberculosis* samples. When mutations are mapped onto these trees, the topology can reveal patterns consistent with positive selection, such as the repeated emergence of mutations across distinct lineages, clustering on terminal branches, or association with rapidly expanding clades [10]– [12]. These patterns suggest that certain positively selected mutations confer a fitness advantage, such as increased transmissibility or resistance to drugs. Notably, such mutations may arise independently in different lineages but are consistently retained, reflecting strong and recurrent adaptive pressures acting on the pathogen population. Identifying these mutations offers an opportunity to uncover the genetic basis of adaptation, revealing both known and previously uncharacterised functional loci that contribute to pathogen persistence and spread.

Graph neural networks (GNNs) are a class of models designed to operate directly on structured graph data. By representing phylogenetic trees as computational graphs, GNNs are uniquely positioned to incorporate both local mutational features and the global topology of evolutionary relationships. In our setting, each node represents an isolate, edges encode phylogenetic neighbourhood relationships, and the node feature indicates the presence or absence of the focal SNP. The model then produces a graph-level prediction for that SNP by learning how its distribution is structured across the phylogeny. Through iterative message passing, these models can learn context-dependent representations that reflect how mutations are embedded within their phylogenetic landscape [13]. This allows the model to ask whether a variant is concentrated in local clades, repeatedly emerges across the tree, or follows other topological patterns associated with selection. A graphical convolutional neural network (GCN) is a classical formulation of the GNN architecture, in which each node aggregates features from its connected neighbours and passes this aggregated signal through neural layers to extract localised information. However, standard GCNs assign uniform or fixed weights to neighbouring nodes, potentially underutilising informative signals in uneven or heterogeneous graph structures. To address this limitation, Graph Attention Networks (GATs) [14] introduce an attention mechanism that allows the model to learn the relative importance of each neighbouring node during message passing. In this framework, attention coefficients are computed dynamically and used to weight each neighbour’s contribution, enabling the network to focus more strongly on structurally or mutationally relevant regions of the phylogeny. This selective aggregation is particularly advantageous in evolutionary graphs, where certain branches or recurrent mutations may carry disproportionately informative signals of adaptation [15].

Here, we investigate the application of GNNs to the task of detecting positively selected variants in *M. tuberculosis*, using SNP-annotated phylogenetic trees as input. This differs from recent machine-learning approaches that classify mutations or genomic profiles according to drug-resistance phenotype, as our objective is to detect signals of positive selection from the phylogenetic distribution of individual variants. Our approach encodes both tree structure and mutational data into a unified graph model, enabling the classifier to distinguish adaptive mutations and those likely to arise under neutral evolution and be passed on due to simple transmission.

## System and methods

### Data structure design and transformation

**Fig. 1:**
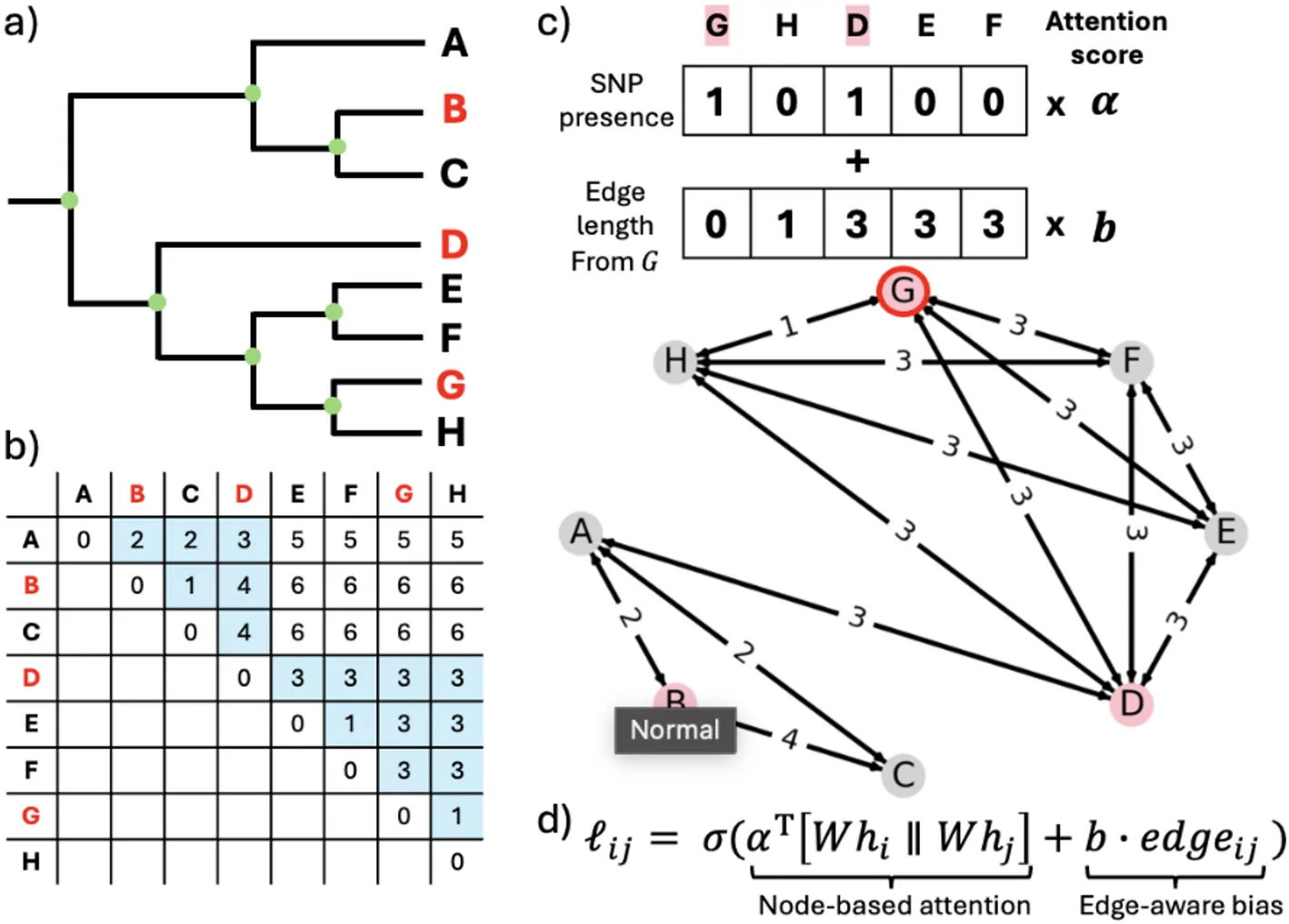
Phylogeny-informed graph construction and node feature propagation using attention-based graph neural networks. **a** Simulated phylogenetic tree of eight samples, internal nodes coloured green, and mutation presence coloured red. **b** Corresponding pairwise phylogenetic distance (internal node count) matrix, where entries denote branch-length distances; blue cells represent closely related clades selected for subgraph construction after > 4 inner node distance cut-off. **c** Edge weights correspond to phylogenetic distances. Attention is computed using both node features (SNP presence) and edge lengths (branch distances). **d** Edge-aware attention score computation in the GAT layer. The raw attention score $\:{\mathcal{l}}_{ij}$ for the edge from node * j* to node * i* is computed from both node features and phylogenetic edge information. Node-based attention is obtained by applying a learnable vector $\:\alpha\:$ to the concatenated linear projections of source and target node features $\:\left[{Wh}_{i}\right|\left|{Wh}_{j}\right]$, while phylogenetic distance is incorporated as an additive edge-aware bias through a learnable parameter * b* and the edge length $\:{edge}_{ij}$. The combined score is passed through a non-linear activation function $\:\alpha\:$ (LeakyReLU) prior to SoftMax normalisation and neighbour aggregation during message passing

To evaluate the capability of graph neural networks in processing phylogenetic tree-structured data, we implemented a GAT model on SNP-annotated *M. tuberculosis* phylogenies. This proof-of-concept aims to assess whether GATs can effectively leverage phylogenetic context to identify functionally relevant SNP patterns across related samples. As illustrated in the simulated tree (Fig. 1a), a SNP appears in samples B, D, and G. Pairwise phylogenetic distances are calculated by counting the number of internal nodes separating each sample (Fig. 1b). In this illustrative example, edges between samples separated by more than four internal nodes were removed to highlight local phylogenetic structure. For real datasets, the cutoff was selected empirically to balance graph sparsity and information retention. More specifically, each isolate was represented as a node, edges were defined between isolate pairs whose phylogenetic distance fell below the chosen threshold, edge attributes stored the corresponding internal-node distance, and the node feature encoded the presence or absence of the focal SNP. This sparsification step was crucial to negate any noisy long-range interactions and emphasise meaningful local neighbourhoods within the tree topology. Without such pruning, the graph would become extremely dense, with each isolate connected to many distant relatives. Although phylogenetic distances would still be retained as edge attributes, message passing in this setting would mix local and long-range information at every layer, potentially diluting the local evolutionary patterns that are most informative for the focal SNP. We therefore used the cutoff as a graph-sparsification step to preserve local neighbourhood structure. The selected value of 7 should be interpreted as an empirically chosen transformation parameter rather than a biologically fixed threshold. Accordingly, this cutoff is specific to the present dataset and graph-construction scheme, and would need to be re-evaluated for phylogenies with different sampling densities or topological structure. The resulting subgraph (highlighted in blue in Fig. 1b) preserves a clear dual-cluster structure, which is also reflected in the corresponding graph visualisation (Fig. 1c). The updated representation of node *i* (denoted as $\:{h}_{\:i}^{{\prime\:}}$) is obtained by combining the attention-weighted features of its connected neighbours (Fig. 1c). Each node in the graph was initialised with a binary feature vector indicating the presence or absence of the target SNP, and this is summed with an edge-aware bias.

In a more generalised equation (Fig. 1d), for a target node * i*, the updated embedding $\:{{h}^{{\prime\:}}}_{i}$ is computed by aggregating messages from its neighbours * j*. The attention score is calculated using a learnable weight vector $\:\alpha\:\:\in\:{\mathbb{R}}^{{F}^{{\prime\:}}}$, applied to the concatenated transformed features of the target node $\:{h}_{i}$ and its neighbour $\:{h}_{j}$, each projected via a shared linear transformation $\:{W\mathbb{\:}\in\:\mathbb{R}}^{{F}^{{\prime\:}}+F}$. The concatenated representation $\:\left[W{h}_{i}\parallel\:W{h}_{j}\right]$ denotes node-pair interactions. Edge information is integrated via a scalar product between a learnable bias parameter $\:b\:\in\mathbb{\:}\mathbb{R}$ and the edge length, $\:{edge}_{ij}$, representing the phylogenetic distance (internal node count) between nodes * i* and * j*. The full attention coefficient is then passed through a non-linear activation function $\:\sigma\:$, here Leaky ReLU (Rectified Linear Unit), to introduce non-linearity before SoftMax normalisation and attention-weighted message aggregation.

### Data sources

The genomic data were derived from transmission records of a multidrug-resistant (MDR+) enriched subset of the in-house 170 K *M. tuberculosis* dataset, collected from publicly available sources [16]. A total of 1,000 *M. tuberculosis* clinical isolates were selected to provide representation across the four major lineages (L1-L4) and a tractable proof-of-concept dataset, rather than as a simple random sample of the full collection. A rooted genome-wide phylogeny was first reconstructed from the 1,000 *M. tuberculosis* isolates, and each focal SNP was then mapped onto this shared tree. The focal SNP was then mapped onto this tree and converted into a graph representation in which leaves corresponded to isolates, pairwise phylogenetic distances were calculated as the number of internal nodes separating isolate pairs, and edges were retained only for pairs within the selected distance threshold. These phylogenies were then transformed into graphs, where nodes represented individual genome samples, and edges reflected undirected phylogenetic connections. Node features were encoded as a single binary variable denoting the presence or absence of the focal SNP. In total, 206 SNP-specific graphs were generated, each corresponding to one focal SNP mapped onto the shared phylogeny, with labels assigned according to WHO catalogue classifications of drug resistance (https://github.com/GTB-tbsequencing/mutation-catalogue-2023/tree/main/Final%20Result%20Files). To implement the model, we applied it to a set of 138 mutations currently classified by the WHO as having “uncertain” associations with drug resistance. Graphs were constructed using PyTorch Geometric [17] and batched using its native DataLoader. The visualisation of the phylogenetic tree was performed using networkX [18] and iTOL [19] tools.

### Bioinformatics pipeline

The raw sequence reads were trimmed using trimmomatic [20] according to the sequence quality generated using fastQC [21]. The trimmed reads were then mapped to the H37Rv reference genome using BWA-mem [22]. SNPs were called using the BCF/VCF [23] tool suite on regions with > 10-fold read depth coverage. SNPs were converted into a FASTA format alignment, which was used as input to RAxML [24] to reconstruct the phylogeny. The resulting tree was rooted using midpoint rooting. Reads were trimmed with Trimmomatic (v0.39) using LEADING:3 TRAILING:3 SLIDINGWINDOW:4:20 MINLEN:36 and mapped with BWA-MEM (v0.7.17-r1188). Variants were called with samtools/bcftools using a minimum read depth threshold of 10. Phylogenies were reconstructed with RAxML (v8) under the GTRGAMMA model. All drug resistance mutations were excluded when building the tree to prevent data leakage.

### Homoplasy-count calculation

To quantify the degree of homoplasy for each SNP, we computed the homoplasy-count using the Fitch parsimony algorithm implemented in Python [25]. For each SNP, an individual phylogenetic tree was constructed from the whole-genome alignment, and the number of independent mutational events required to explain the observed distribution of alleles across the tree was recorded as its homoplasy-count. Variants with higher scores indicate recurrent emergence across distinct lineages, consistent with convergent evolution or positive selection, whereas lower scores correspond to lineage-specific or clonal variants. A threshold of three independent occurrences (homoplasy-count ≥ 3) was used as an empirical cutoff for the homoplasy-count baseline. This threshold was chosen as a practical comparator against curated labels rather than as a formally significance-calibrated test of recurrent mutation. As a qualitative assessment, our results were compared with genes under positive selection identified by *M. tuberculosis* studies using PAML [5] and PhyC methods [4].

### Statistical comparisons

To assess whether the GAT and the homoplasy-count baseline differed in performance, we used two complementary procedures. First, on the held-out test set (*n* = 42), we applied McNemar’s exact test to their paired per-variant predictions. This test evaluates the discordant cases - variants classified correctly by one method but not the other - and is equivalent to testing whether the two methods differ in overall accuracy on the same samples. Because a single held-out split provides limited statistical power at this sample size, we additionally performed repeated stratified k-fold cross-validation over the full set of 206 variant graphs (five folds, five repeats, yielding 25 independent train/test partitions; stratification preserved class balance within each fold). In each fold, the GAT was retrained from scratch using the architecture and hyperparameters described in Sect. 3.1, and both methods - the GAT and the homoplasy-count baseline (homoplasy count ≥ 3) - were evaluated on the held-out portion of that fold. Per-fold accuracy and F1 were summarised as means with 95% confidence intervals across the 25 folds, and the two methods were compared using the paired Wilcoxon signed-rank test on per-fold scores. All analyses used a fixed master seed (42), with per-fold seeds derived deterministically to ensure reproducibility.

## Algorithm

### GNN architecture and training

To classify SNP presence using phylogenetically structured data, we employed Bayesian hyperparameter optimisation to identify the optimal configuration for model training. The final architecture was implemented using PyTorch Geometric (v2.4.0)^17^ and consisted of a two-layer Graph Attention Network (GAT) (**Table ****S1**). Edge-level features (phylogenetic edge lengths) were used as scalar edge lengths during message passing and were supplied to both GATConv layers via the edge_attr argument. The first GATConv layer used 8 attention heads, each producing 32 output features, resulting in a concatenated 128-dimensional output. This was followed by batch normalisation, Exponential Linear Unit (ELU) activation, and dropout (*p* = 0.29). The second GATConv layer maintained the same dimensionality (128 features) with a single attention head and concat=False to allow residual connection with the first layer. The output of this layer was batch-normalised, passed through an ELU activation, and added to the first layer’s output via a residual connection. Dropout was applied after both GAT layers.

For graph-level prediction, node embeddings were aggregated using a global attention pooling layer with a linear gating function to compute attention scores across nodes. The resulting pooled feature vector was passed through a fully connected layer with 32 hidden units, followed by batch normalisation, ReLU activation, and dropout. A final linear layer projected the representation to a two-dimensional output space for binary classification. Class probabilities were obtained by applying a SoftMax function over the two logits. The probability (* p*) corresponding to the predicted class was then passed through a sigmoid transformation to yield a confidence score between 0 and 1. For negative predictions (probability < 0.5), the confidence was defined as * 1-p*, ensuring that the reported value consistently reflects the model’s certainty in its assigned label.

Training was conducted using the Adam optimiser in conjunction with a one-cycle learning rate policy.

The learning rate was annealed from a minimum of 9.89 × 10⁻⁵ to a maximum of 7.55 × 10⁻⁴ using a cosine annealing schedule. The loss function used was cross-entropy with class weights of 1.0 for negative cases and 1.17 for positive cases to address mild class imbalance. The model was trained for a maximum of 700 epochs, with early stopping applied based on validation loss plateauing; in all runs, the stopping criterion was reached before the maximum epoch limit. For supervised evaluation, the 206 mutation graphs were partitioned into training (123), validation (41), and held-out test (42) sets.

### Edge attention

Attention coefficients in a GAT allow a node to adaptively weight information from its neighbours, capturing their varying importance. Attention coefficients were obtained from the GAT using the PyTorch Geometric GATConv layers. Unless stated otherwise, attention was extracted from the first GAT layer. Multi-head coefficients were averaged across heads to yield a single edge-level score. Attention scores were aligned to the undirected NetworkX graph used for visualisation by matching both (u, v) and (v, u) tuples. Undirected graphs were rendered with NetworkX using a spring layout. Nodes were coloured by mutation presence, and edges were coloured by attention weights. To quantify how concentrated the model’s focus is, the Top-k Attention Mass (TAM) was computed, $\:TAM\left(k\right)=\:\frac{{\sum\:}_{i=1}^{m}{\alpha\:}_{\pi\:\left(i\right)}}{{\sum\:}_{e=1}^{E}{\alpha\:}_{e}}\:,$ where $\:\alpha\:={\left\{{\alpha\:}_{e}\right\}}_{e=1}^{E}$ denotes the averaged attention scores for the E edges. Sorting in descending order gives indices π such that $\:\alpha \:_{{\pi \:\left( 1 \right)}} \ge \:\alpha \:_{{\pi \:\left( 2 \right)}} \ge \:1/4.$ For a chosen fraction $\:k\in\:\left(\mathrm{0,1}\right],\:m=\:kE$ edges are retained.

## Implementation

### GAT training with supervised learning

**Fig. 2:**
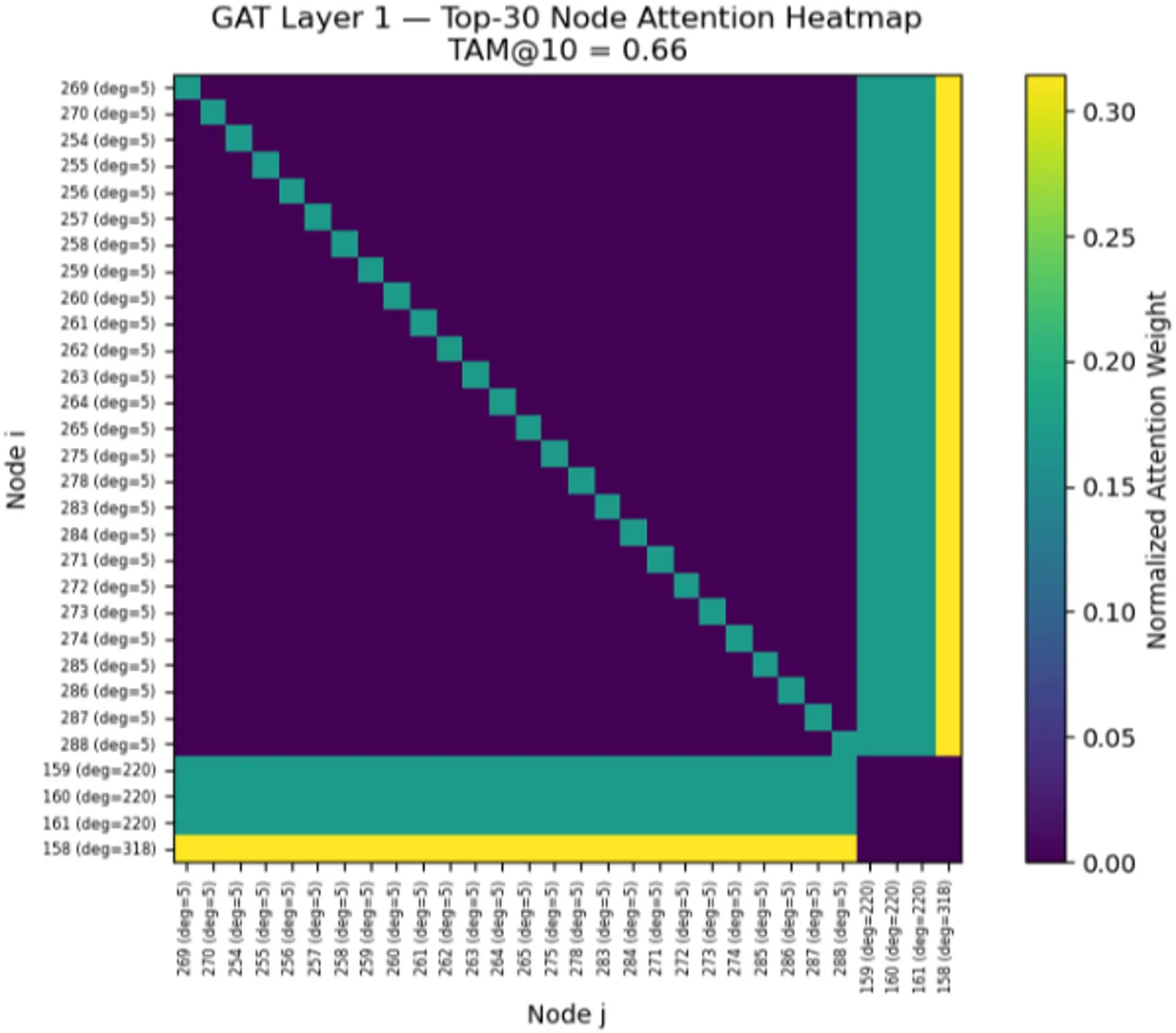
Edge-level attention weights. Heatmap showing how much attention the model pays to different transformed edge connections of the phylogenetic tree when looking at the *rpoB* Ser450Leu rifampicin resistance mutation, as measured by the weights in the first layer of the GAT. High attention is coloured yellow, while low attention is coloured blue. Axis tick values show the node index and degree (deg): number of edges connected to the node

The dataset used in this study comprises 1000 *M. tuberculosis* samples, spanning the four main lineages (*n*: L1 23, L2 578, L3 72, L4 327), and 206 SNP variants identified in 79 drug resistance genes. Among these, 75 mutations (36.4%) were annotated by the WHO as being associated with drug resistance, while 131 were classified as not associated with resistance. A total of 206 graph instances (1,000 nodes) were generated, each corresponding to a single variant. Each graph shared a common underlying topology derived from a simplified phylogenetic tree, with node labels indicating the presence or absence of the specific mutation under consideration. The graphs were simplified by removing edges between sample pairs separated by more than seven internal nodes in the phylogenetic tree (cut-off: edge length > 7), leading to more interpretable network structure reflecting localised phylogenetic neighbourhoods. For example, for the *rpoB* mutation Ser450Leu mutation linked to rifampicin resistance, the pruning revealed two distinct clusters connected by a small number of high-degree nodes acting as bridges (**Figure ****S1**). The GAT assigns disproportionately close attention to edges connecting central nodes, forming a dense hub of influential connections (Fig. 2). This pattern is consistent with a TAM distribution, in which the highest-ranked 10% of edges (k = 10%) capture 65.6% of total attention.

### GAT predictive performance

The GAT models demonstrated strong predictive performance when trained on the graph-encoded phylogenetic structure. On the held-out test dataset comprising 42 variant graphs (15 under positive selection and 27 neutrally evolving), the GAT achieved an accuracy of 0.81, an area under the ROC curve (AUC) of 0.82, and an F1 score of 0.71, with a sensitivity of 0.67 and a specificity of 0.89. These results underscore the model’s ability to distinguish positively selected mutations from neutral variants in a topology-aware manner (Table 1). Of the 42 variants in the test set, 20 were non-synonymous mutations (1 start-lost and 19 missense), 14 were synonymous mutations, 1 was annotated as a non-coding transcript exon variant, and 7 were upstream gene variants. As a supplementary sensitivity analysis, we re-evaluated the held-out predictions on the 20 coding nonsynonymous variants in the test set. On this subset, the GAT achieved an accuracy of 0.90, precision of 1.00, sensitivity of 0.78, specificity of 1.00, and F1 score of 0.88.

**Table 1:** Confusion matrix of GAT model predictions of 42 SNP classifications in comparison to baseline

GAT*		Predicted Neg	Predicted Pos
	Actual Neg	TN = 24	FP = 3
	Actual Pos	FN = 5	TP = 10
Homoplasy-count baseline**		Predicted Neg	Predicted Pos
	Actual Neg	TN = 16	FP = 11
	Actual Pos	FN = 1	TP = 14

The matrix summarises the performance of the graph attention network and homoplasy baseline method on the test (hold-out) dataset

*Accuracy 0.81, Precision 0.77, Sensitivity 0.67, Specificity 0.89, AUC 0.82, F1 0.71

**Accuracy 0.71, Precision 0.56, Sensitivity 0.93, Specificity 0.59, AUC 0.64, F1 0.70

**Fig. 3:**
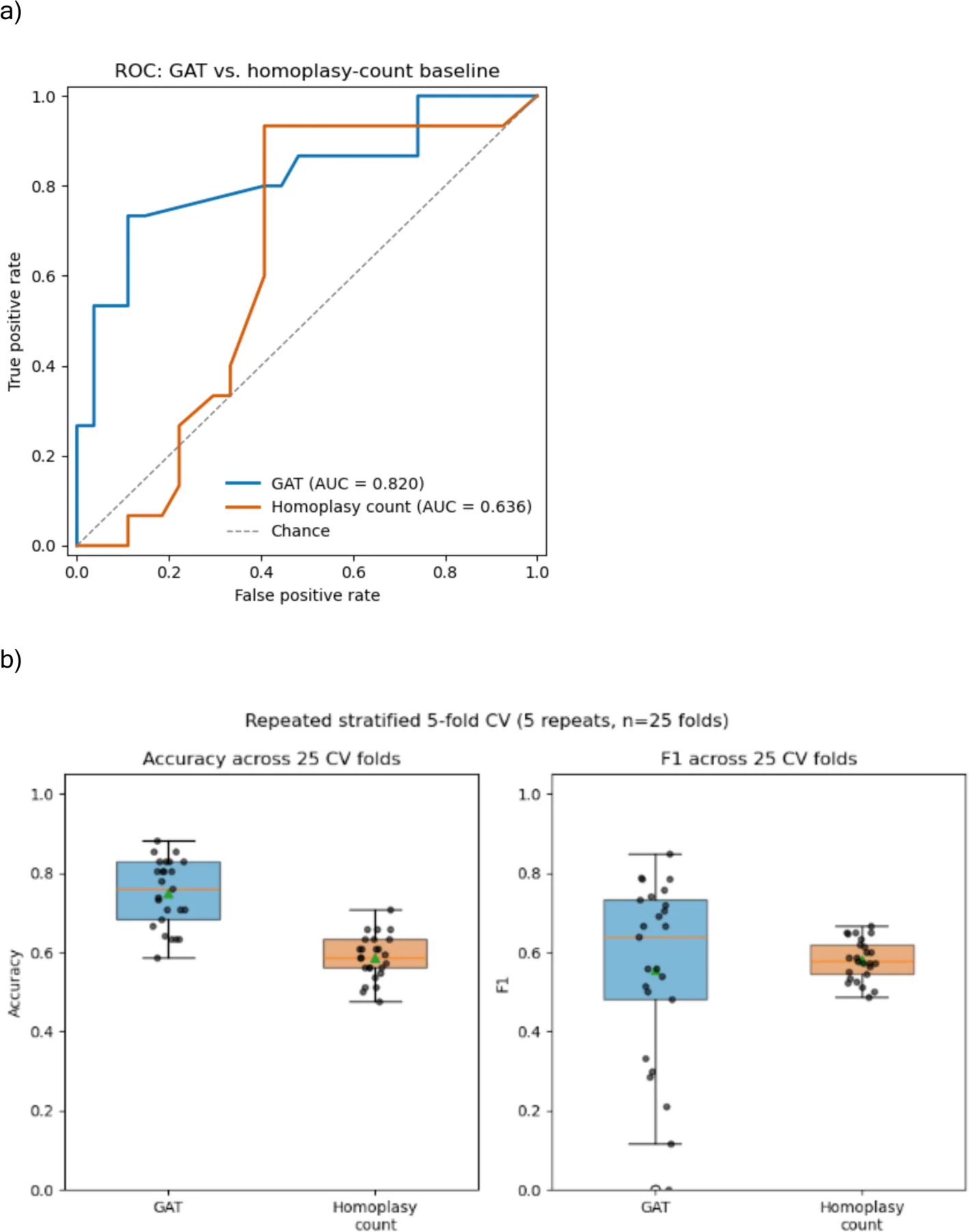
Comparison of the graph attention network (GAT) and the homoplasy-count baseline for detecting positive selection. **a** Receiver operating characteristic (ROC) curves on the held-out test set (*n* = 42), using the GAT predicted probability and the continuous homoplasy count as classifier scores. The GAT achieved a higher area under the curve (AUC = 0.82) than the homoplasy-count baseline (AUC = 0.64), indicating stronger threshold-free discrimination. **b** Per-fold accuracy and F1 across repeated stratified five-fold cross-validation (five repeats, 25 folds) over all 206 variants. The GAT showed significantly higher accuracy (paired Wilcoxon *p* < 0.001) but statistically indistinguishable F1 (*p* = 0.98) relative to the baseline, consistent with a sensitivity-specificity trade-off. Boxes show the median and interquartile range, whiskers extend to 1.5x the IQR, and points show individual folds, including four folds in which the GAT F1 fell below 0.3

We compared the GAT with a homoplasy-count baseline (Fitch parsimony, cutoff ≥ 3). On the held-out test set, the baseline showed higher sensitivity (0.93 vs. 0.67) but lower specificity (0.59 vs. 0.89), lower accuracy (0.71 vs. 0.81), and lower ROC AUC (0.64 vs. 0.82; Fig. 3a), with near-identical F1 (0.70 vs. 0.71). The accuracy difference was not significant (McNemar’s exact test, *p* = 0.45). Under repeated stratified five-fold cross-validation (five repeats, 25 folds) over all 206 variants (Fig. 3b), the GAT achieved higher mean accuracy (0.75, 95% CI 0.71–0.78 vs. 0.59, 95% CI 0.56–0.61; Wilcoxon *p* < 0.001) but statistically indistinguishable F1 (0.56, 95% CI 0.46–0.65 vs. 0.58, 95% CI 0.56–0.60; *p* = 0.98). Both evaluations point to the same sensitivity-specificity trade-off, with the GAT the more specific classifier and the baseline the more sensitive one, rather than either being uniformly superior. Stratifying by homoplasy status, all 10 held-out GAT true positives were homoplasic, as were 4 of the 5 false negatives; only *pncA* Pro54Leu (homoplasy count = 1) was a non-homoplasic false negative. Of the 13 GAT-positive calls, 11 were also flagged by the baseline; the two exceptions - *rpoC* c.186 C > T and *fbiA* c.337 C > T - had homoplasy counts of 2.

Among the variants predicted by the model to be under positive selection, 7 were missense mutations, 2 were upstream promoter variants, 2 were synonymous substitutions, and 1 was a non-coding transcript exon variant (*rrs* n.514 A > C). Notably, synonymous mutations such as *embB c.1602 C > T* and *fbiA c.337 C > T* are not typically associated with functional change or drug resistance (ethambutol, pretomanid, delamanid, and clofazimine) and are therefore unlikely to be true targets of selection. The near-complete focus of the model on nonsynonymous or regulatory variants supports its capacity to prioritise biologically meaningful signals of adaptation, highlighting its potential to capture genomic features relevant to antimicrobial resistance. Because the benchmark includes upstream, synonymous, and non-coding variants in addition to coding nonsynonymous SNPs, direct comparison with codon-based methods such as PAML, pNpS or Ka/Ks is not straightforward in the current setting. However, 29 of the 42 SNPs (69.0%) were located in genes identified as being under positive selection [4], [5] (**Table ****S2**).

### Testing on uncertain mutations

To evaluate the model’s utility beyond annotated resistance mutations, we tested it on 138 candidate variants classified as “uncertain” in the WHO catalogue. Of these, 44 (31.9%) were predicted by the model to be under positive selection, with 28 being high-confidence (scores > 0.54) predictions (**Table S3**). These included putative drug resistance and compensatory mutations, such as *rpoC* (Val483Ala, Glu1033Lys) (rifampicin, compensatory [26]), *ubiA* (Val188Ala, Arg240Cys, Ala249Thr) (ethambutol), *rpsA* Ala412Val (Pyrazinamide), *Rv1979c* Arg409Gln (clofazimine, bedaquiline), and *whiB6* Ala66Thr. The *ubiA *gene encodes a protein essential for cell wall synthesis and is implicated in ethambutol resistance. The *rpoC* encodes the β subunit of RNA polymerase, where mutations often compensate for fitness costs associated with *rpoB*-mediated rifampicin resistance. The *rpsA* encodes ribosomal protein S1, a target of pyrazinoic acid, and its mutations have been associated with pyrazinamide resistance. The *whiB6* coordinates the bacteria’s intrinsic and acquired drug resistance mechanisms. Evidence for positive selection is further supported by the absence of these mutations in drug-sensitive strains and their presence in MDR strains, where they occur at frequencies exceeding 17% across at least 11 sublineages in the 100 K dataset. The only exceptions are Rv1979c Arg409G and Rv1258c Val245Met (**Table S3**).

## Discussion

TB remains a leading global health threat, fuelled by the emergence of drug-resistant strains. This study explores the application of GATs to detect signals of positive selection in *M. tuberculosis* phylogenies, which can serve as genomic signatures of drug resistance, but may also be driven by mutations that enhance bacterial survival or transmission without directly conferring drug resistance. This interpretation can be complex; for example, the *whiB6* locus may be linked not only to acquired and intrinsic drug resistance, but also to virulence through its regulation of ESX-1 gene expression, which in turn controls the secretion of virulence factors. By focusing on well-characterised drug-resistance and neutral mutations, the GAT showed promising performance despite the small and imbalanced dataset, with accuracy exceeding 80% and evidence of across-lineage recurrence. Further, a deeper examination of the attention weights in the edges revealed that the model was capable of selectively focusing on the most informative regions of the graph, while attenuating attention toward genetically uniform areas. This behaviour suggests that the model is able to prioritise informative regions of the graph. It also suggests that the use of internal node counts as edge lengths for converting phylogenies into graph neural network (GNN)-trainable data, simplifies the phylodynamic graph structure while preserving essential information, thereby supporting its novel application for the detection of positive selection.

Importantly, the model does not explicitly implement a codon-based selection test or a simple homoplasy-count rule. Rather, it treats the phylogeny-derived graph and the SNP distribution across isolates as input to a graph-classification model, allowing recurrent emergence and local phylogenetic structure to be learned jointly rather than reduced to a single summary statistic. Compared with the homoplasy-count baseline, the GAT yielded a more specific but less sensitive classifier on the held-out test set. This suggests that graph-based learning may be useful for prioritisation tasks where false positives are costly, although larger-scale benchmarking against existing selection methods is needed. Unlike the homoplasy-count baseline, which reduces each SNP to the number of inferred independent mutation events, the GAT can also use local phylogenetic neighbourhood structure, including clustering of mutation-bearing isolates and the arrangement of mutation-positive and mutation-negative samples across nearby branches.

The GAT approach was also applied to mutations of uncertain significance and identified a set of candidate variants that may contribute to *M. tuberculosis* spread through drug resistance or enhanced bacterial fitness. Reassuringly, the model highlighted additional putative resistance and compensatory mutations, almost all of which were absent from drug-sensitive strains and present in a global set of MDR strains. Many of these variants show convergent emergence across resistant lineages, such as *embA* c.-16 C > A, *ubiA* Ala249Thr, *mmpS5* c.-74G > T, *whiB6* Ala66Thr, and *rpoC* Ile491Val, underscoring their likely functional relevance. However, as illustrated by the pleiotropic effects of the *whiB6* locus discussed above, positive selection acting on these mutations may reflect not only drug resistance but also broader fitness advantages. Overall, these findings suggest that the model can flag previously unclassified variants for closer scrutiny, helping to prioritise experimental and epidemiological studies aimed at disentangling their roles in resistance and transmissibility. From a technical perspective, while the model may correctly identify such variants, mislabelling during training could introduce noise, reduce training efficiency, and impose an upper limit on predictive performance.

A further limitation is that each graph instance represents a single focal SNP, encoded only by its presence or absence across isolates. The model therefore does not explicitly capture epistatic interactions, such as compensatory or stepping-stone effects, or directly estimate lineage-specific effects on drug resistance. Extending the framework to include multi-variant node features or joint mutational context would be an important next step. Moreover, as with human interpretation of phylogenetic trees, model performance depends on the number of truly positively selected mutations and the size of the dataset. In this context, our analysis of the “uncertain” section of the WHO catalogue provides an important demonstration of the model’s broader utility.

Nonetheless, this proof-of-concept study was based on a relatively limited dataset of 206 SNP-specific graphs sharing a common phylogenetic topology. Expanding to larger cohorts with greater genetic diversity is expected to strengthen predictive power and improve generalisation. To our knowledge, this is an early application of graph neural networks to SNP-level positive-selection prediction on *M. tuberculosis* phylogenies, illustrating the potential of aligning phylogenetic data representation with model architecture. Future work could involve adapting the architecture for graph-agnostic learning, allowing the model to transfer knowledge across diverse phylogenetic contexts and potentially detect adaptive signals in a wider range of pathogens. Applied to *M. tuberculosis*, such approaches could yield deeper insights into the evolutionary dynamics of mutations that enhance bacterial survival or broader forms of drug resistance, ultimately informing strategies for improved disease control and treatment.

## Supplementary Information

Below is the link to the electronic supplementary material.

Supplementary Material 1

Supplementary Material 2

## Data Availability

The sequencing data analysed in this study are publicly available from the European Nucleotide Archive (ENA). No new biological samples were collected, and no new primary sequencing data were generated as part of this study. The complete list of study, sample, and run accession numbers corresponding to all isolates included in the analysis is available as a CSV file in the as supplementary data. The full codebase for preprocessing, model training, and evaluation, are available at: https://github.com/linfeng-wang/Phylogeny-Guided_GAT. These resources enable full reproducibility of the results presented in this study.
