# Supplementary material for: Decoding positive selection in Mycobacterium tuberculosis with phylogeny-guided graph attention models
Source: BMC Bioinformatics. 2026 Aug 6;27:261. doi: 10.1186/s12859-026-06583-0 (PMC13733096; doi:10.1186/s12859-026-06583-0)
Supplement: Supplementary file 1 [file 12859_2026_6583_MOESM2_ESM.docx]

**SUPPLEMENTARY INFORMATION**

### **Figure S1. SNP-based phylogenetic tree instance and its associated Graph Attention Network (GAT) model (n=1,000) for the rifampicin resistance**

### (A) **Phylogenetic tree for the *rpoB* mutation Ser450Leu** (Mutation presence - red; Mutation absence – grey)

(B) **GAT input graph for (A).** This graph structure (the *rpoB* - Ser450Leu) is used as input for GAT training, allowing the model to propagate and weight information through biologically relevant neighbours. (Mutation presence - red; Mutation absence – grey)

**Table S1. Model architecture**

| **Stage** | **Layer** | **Heads** | **Concat** | **Output shape** |
| --- | --- | --- | --- | --- |
| Node feats | Input  (node feature-SNP presence; Edges-node connection; Edge-length) | – | – | Node×1 |
| Message pass 1 | GATConv (out=32 per head) | 8 | Yes | Node×(32x8=256) |
| Norm/act | BN → ELU → Dropout | – | – | Node×256 |
| Message pass 2 | GATConv (out=256, head=1) | 1 | No | Node×256 |
| Residual | BN(x₂) + x₁ → ELU → Dropout | – | – | Node×256 |
| Readout | GlobalAttention (gate: 256→1) | – | – | Batch×256 |
| MLP | FC(256→32) → BN → ReLU → Dropout → FC(32→2) | – | – | Batch×2 |

BN: batch normalisation; ELU: Exponential Linear Unit (ELU) function; FC: Fully connected linear layer; ReLu: Rectified Linear unit

**Table S2. Test set predictions from the Graph Attention Network (GAT) Models across the 42 variants***

| **Gene** | **SNP mutation** | **Parsimony Score**** | **Label*** | **Prediction** | **Positive selection***** |
| --- | --- | --- | --- | --- | --- |
| *embB* | met306val | 62 | 1 | 1 | Yes |
| *rrs* | n.514a>c | 17 | 1 | 1 | Yes |
| *pncA* | c.-11a>g | 12 | 1 | 1 | Yes |
| *embB* | gly406asp | 10 | 1 | 1 | Yes |
| *pncA* | _p.met1? | 7 | 1 | 0 | Yes |
| *eis* | c.-37g>t | 5 | 1 | 0 | Yes |
| *ethA* | c.-7t>c | 4 | 1 | 0 | Yes |
| *pnca* | trp68arg | 4 | 1 | 1 | Yes |
| *inhA* | c.-770t>a | 4 | 1 | 0 | Yes |
| *rpoB* | his445arg | 3 | 1 | 1 | Yes |
| *inhA* | c.-779g>t | 3 | 1 | 1 | Yes |
| *gyrB* | ser447phe | 3 | 1 | 1 | Yes |
| *pncA* | val180phe | 3 | 1 | 1 | Yes |
| *rpoB* | ile491phe | 3 | 1 | 1 | Yes |
| *pncA* | pro54leu | 1 | 1 | 0 | Yes |
| *whib6* | c.-75delg | 132 | 0 | 0 | Yes |
| *tsnr* | leu232pro | 129 | 0 | 0 | Yes |
| *gyrA* | gly668asp | 129 | 0 | 0 | Yes |
| *ahpC* | c.-88g>a | 24 | 0 | 0 |  |
| *rpoB* | c.2628t>g | 24 | 0 | 0 | Yes |
| *rpoC* | glu1092asp | 17 | 0 | 0 | Yes |
| *glpK* | c.510c>t | 7 | 0 | 0 |  |
| *rv3236c* | ala370thr | 7 | 0 | 0 |  |
| *rpoB* | c.309c>t | 6 | 0 | 0 | Yes |
| *gyrB* | c.1578g>a | 4 | 0 | 0 | Yes |
| *embB* | c.1602c>t | 4 | 0 | 1 | Yes |
| *ubiA* | c.294c>t | 2 | 0 | 0 |  |
| *ubiA* | c.228t>c | 2 | 0 | 0 |  |
| *ppe35* | gly258asp | 2 | 0 | 0 |  |
| *rpoC* | pro601leu | 2 | 0 | 0 | Yes |
| *rpoC* | c.186c>t | 2 | 0 | 1 | Yes |
| *gyrB* | val301leu | 2 | 0 | 0 | Yes |
| *ndH* | val18ala | 2 | 0 | 0 |  |
| *rv1979c* | asp286gly | 2 | 0 | 0 |  |
| *rv0010c* | ile87met | 2 | 0 | 0 |  |
| *embC* | asn394asp | 2 | 0 | 0 | Yes |
| *rv3083* | c.105g>a | 2 | 0 | 0 |  |
| *gid* | c.330g>t | 2 | 0 | 0 | Yes |
| *rpoC* | c.517c>a | 2 | 0 | 0 | Yes |
| *fbiA* | c.337c>t | 2 | 0 | 1 |  |
| *rpoA* | c.972c>t | 1 | 0 | 0 |  |
| *lpqB* | c.1689a>g | 1 | 0 | 0 |  |

* 15 under positive selection (label = 1) and 27 neutrally evolving (label = 0); ** from the Fitch parsimony algorithm; *** Genes identified in Deng et al, 2024 (doi: 10.3389/fmicb.2024.1290227) or Farhat et al, 2013 (doi: 10.1038/ng.2747)

**Table S3. WHO Drug resistance Uncertain variants predicted to be under positive selection with confidence scores of at least 0.55**

| **Gene** | **Change** | **Confidence** | **MDR+ (%)*** | **No. Sub-Lin.**** | **Prominent Lineage (freq. %)***** | **Notes** |
| --- | --- | --- | --- | --- | --- | --- |
| *rpoC* | Val483Gly | 0.99 | 4513 (49.4) | 37 | 2.2.1 (33.0) | Compensatory^19^ |
| *rpoC* | Lys445Arg | 0.99 | 328 (49.1) | 32 | 2.2.1 (63.0) | Compensatory^27^ |
| *rpoC* | Ile491Val | 0.99 | 1848 (49.5) | 44 | 2.2.1 (67.0) | Compensatory |
| *rpoC* | Glu1033Lys | 0.95 | 109 (49.1) | 22 | 2.2.1 (46.0) | - |
| *ubiA* | Arg240Cys | 0.94 | 135 (48.7) | 28 | 4.6.1.2 (49.0) | Putative^28^ |
| *rpoC* | Asp485Tyr | 0.92 | 705 (49.7) | 32 | 2.2.1 (79.0) | Compensatory^23^ |
| *Rv1979c* | Arg409Gln | 0.76 | 0 (-) | 0 | 2.2.1 (-) | Putative^18^ |
| *rrs* | n.1489C>T | 0.75 | 9 (39.1) | 11 | 2.2.1 (36.0) | - |
| *ubiA* | Ala249Thr | 0.75 | 535 (50.0) | 28 | 2.2.2 (88.0) | - |
| *embA* | c.-16C>A | 0.75 | 467 (49.1) | 41 | 2.2.1 (36.0) | - |
| *ethA* | Pro334Ala | 0.75 | 376 (24.9) | 21 | 2.2.1 (99.0) | - |
| *embA* | c.-8C>A | 0.75 | 347 (49.4) | 34 | 2.2.1 (81.0) | - |
| *rpoC* | Val431Met | 0.75 | 197 (49.0) | 36 | 2.2.1 (43.0) | - |
| *rpsA* | Ala412Val | 0.75 | 196 (42.9) | 14 | 2.2.1 (98.0) | Putative^29^ |
| *whiB6* | Ala66Thr | 0.75 | 192 (48.5) | 11 | 2.2.1 (94.0) | - |
| *katG* | c.-507C>G | 0.74 | 979 (49.57) | 22 | 2.2.1 (98.0) | - |
| *mmpS5* | c.-74G>T | 0.74 | 1557 (46.9) | 27 | 2.2.1 (98.0) | Putative^30^ |
| *Rv1258c* | Val245Met | 0.74 | 0 (-) | 0 | 2.2.1 (-) | - |
| *ubiA* | Val188Ala | 0.71 | 266 (49.7) | 22 | 4.3.3 (84.0) | Putative^24^ |
| *rrl* | n.2771C>T | 0.71 | 257 (17.5) | 48 | 2.2.1 (17.0) | - |
| *rrl* | n.2725A>G | 0.71 | 218 (18.8) | 43 | 2.2.1 (21.0) | - |
| *rrl* | n.2715T>C | 0.71 | 214 (20.1) | 43 | 2.2.1 (21.0) | - |
| *rrl* | n.2726T>A | 0.71 | 180 (20.0) | 49 | 2.2.1 (21.0) | - |
| *rrl* | n.2712C>T | 0.71 | 149 (19.0) | 47 | 2.2.1 (21.0) | - |
| *rrl* | n.2751G>A | 0.71 | 137 (19.2) | 49 | 2.2.1 (16.0) | - |
| *rpoC* | Asn698Ser | 0.64 | 408 (49.6) | 29 | 2.2.1 (70.0) | Compensatory^23^ |
| *rpoC* | Val483Ala | 0.63 | 1570 (49.7) | 33 | 2.2.1 (52.0) | Putative^21^ |
| *rrl* | n.-49G>C | 0.55 | 21 (23.1) | 15 | 3 (94.0) | ^-^ |

* None of the variants are present in the sensitive samples in the 100K dataset; MDR+ indicates the number of samples containing the variant that fall into multidrug-resistant (MDR), pre-extensively drug-resistant (Pre-XDR), or extensively drug-resistant (XDR) categories. % presence refers to the proportion of MDR+ samples in the 100K database that carry the variant. ** represents the number of distinct sublineages in which the variant is found, capturing the breadth of its phylogenetic distribution. *** denotes the sublineage in which the variant is most observed; % Occurrence indicates the percentage of variant-containing samples that belong to this specific sublineage.
